# Anxiety and Depressive Symptoms in Parents of Extremely Preterm Newborns and Their Association with Parent-child Bond

**DOI:** 10.1192/j.eurpsy.2025.1174

**Published:** 2025-08-26

**Authors:** A. Romero Teruel, M. J. Molina-Aguilera, J. Merchán-Naranjo, E. Rodriguez-Toscano, B. Almansa, S. Zeballos-Serrato, D. Blanco-Bravo, A. Garcia-Blanco, L. Pina-Camacho

**Affiliations:** 1 Department of Psychiatry, Hospital Dr. Rodríguez Lafora; 2 Department of Child and Adolescent Psychiatry, Institute of Psychiatry and Mental Health, Hospital General Universitario Gregorio Marañon, CIBERSAM, IiSGM, School of Medicine, Universidad Complutense, Madrid; 3 Neonatal Research Group, Health Research Institute La Fe, University and Polytechnic Hospital La Fe, Valencia; 4 Neonatology Service, Hospital General Universitario Gregorio Marañon, IiSGM, School of Medicine, Universidad Complutense, Madrid, Spain

## Abstract

**Introduction:**

Extreme preterm birth (<28 weeks) is a significant risk factor for adverse neurodevelopmental outcomes in infants but also for heightened psychological stress in parents, both of these impacting on parent-child bonding style. Abnormal parent-child bonding may impact, in turn, on child’s emotional development. Longer stays at NICUs, with their stressful environment and caregiving demands, alongside psychosocial factors and perinatal complications can exacerbate parental emotional stress.

**Objectives:**

(i) To compare, around birthtime, levels of anxiety and depressive symptoms in parents of extremely preterm newborns (EPTN) compared to parents of born-at-term healthy control (HC) newborns; (ii) to assess, in parents of EPTN, longitudinal changes in levels of anxiety and depressive symptoms from birth up to 40 postmenstrual weeks (40PMW), and their association with demographic, clinical and environmental risk factors; (iii) to assess, in parents of EPTN, the impact of NICU-related stress and psychosocial / family context on levels of symptoms; and (iv) to examine, in parents of EPTN, the association between levels of anxiety and depressive symptoms and parent-child bonding quality around hospital discharge.

**Methods:**

Observational, longitudinal, prospective, 24-month follow-up study. We recruited a cohort of n=150 EPTN and n=50 HC (the PeriStress-PremTEA cohort) in two tertiary hospitals in Spain. Of those, parents of n=70 EPTN and n=42 HC successfully completed, around birthtime (and at 40 PMW in EPTN) the STAI state & trait, BDI, PBQ, MSPSS and PPS at NICU questionnaires. We also gathered demographic, clinical and obstetric data from all patient & HC families. We compared, in EPTN vs HC, parental symptom levels around birth. Using logistic regression, we assessed, in EPTN, the association between demographic/perinatal variables, parental symptom levels around birth time and parent-child bonding quality at discharge.

**Results:**

Both fathers and mothers of EPTN showed higher anxiety and depressive symptom levels than those of HC (all p<.01). In EPTN, parental symptom levels around birth time were highly correlated with PSS NICU scores (p<.01). Levels of anxiety and depressive symptoms in mothers of EPTN predicted parent-child bonding quality at discharge, above and beyond other potential risk factors, as did level of depressive symptoms in fathers of EPTN (p<.01).

**Conclusions:**

Our findings support the need to establish screening and longitudinal monitoring programs of psychopathology in parents of EPTN, both for mothers and fathers of these children, and even more so in higher-risk subgroups, such as those with higher perception of NICU-related stress.

**Disclosure of Interest:**

None Declared

